# Comparative Screening of Chlamydia Trachomatis Infection in Women Population in Tehran, Iran

**Published:** 2012-05-30

**Authors:** B Fatholahzadeh, A Bahador, M Haghighi Hasanabad, F Bazarjani, F Haghighi

**Affiliations:** 1 Departments of Microbiology, School of Medicine, Tehran University of Medical Sciences, Tehran, Iran; 2 Molecular and Cellular Biology Research Center, School of Medicine, Sabzevar University of Medical Sciences, Sabzevar, Iran; 3 Department of Microbiology, Faculty of Public Health, Tehran University of Medical Sciences, Tehran, Iran

**Keywords:** Chlamydia trachomatis, Prevalence, Women, PCR, Iran

## Abstract

**Background:**

There are more than 30 different sexually transmissible agents while the most common one is Chlamydia trachomatis. In this prospective study, we decided to compare the prevalence of infection in symptomatic and asymptomatic females.

**Methods:**

Two hundred sixty urine samples of women in two groups (symptomatic and asymptomatic) were collected from patients attending Mehrad Hospital in Tehran, Iran and tested by polymerase chain reaction.

**Results:**

Thirty nine women in both groups were infected (14.99%), while 27/130 subjects were in symptomatic group (20.76%), compared with 12/130 person in asymptomatic group (9.23%). No statistically significant difference was found between two groups. Data analysis showed infection with C. trachomatis in symptomatic women to be significantly associated with history of sexually transmitted infections, white blood cells in urine and epithelial cells in urine.

**Conclusion:**

The present study recommends that targeted screening programs in high risk sexually active women (like as individuals who had a history of STIs) are needed as part of case-finding strategies and treatment.

## Introduction

Sexually transmitted infections (STIs) are the second major cause of unpleasant diseases in young adult women worldwide.[1] Genital infection of Chlamydia trachomatis (C. trachomatis) is one of the most common STIs and is considered as the most common treatable and preventable of them with adverse consequences like infertility and preterm delivery. Most infections in females (60-80%) are asymptomatic, but the disease spectrum includes mucopurulent cervicitis (MPC), endometritis, salpingitis, the urethral syndrome, proctitis, post-abortal pelvic sepsis and perihepatitis.[2] Nowadays, detection of STIs has improved with development of nucleic acid based amplification methods such as polymerase chain reaction (PCR) with >95% sensitivity and specificity for detection of C. trachomatis in urine.[3] The important aspect of these assays is their capacity to be used on non-invasive samples such as first void urine (FVU), which can be self collected and therefore facilitates larger investigations on prevalence in many populations.[4]

It is important to control the spread of C. trachomatis infection, and prevention is the key of this process. Prevention should be based on identification of symptomatic and asymptomatic people, especially those at increased risk for acquisition of new infections.[5] Many developed countries established a national standard plan for screening of C. trachomatis infections[6] but in many hospitals and clinics of Iran, there is no program for the screening of people.

The aim of this study was to determine the prevalence of C. trachomatis infections in 2 groups of females (symptomatic and asymptomatic) and related risk factors in order to accelerate progress towards the strategies of WHO for prevention and control of STIs.[7]

## Materials and Methods

Two hundred and sixty urine samples of females who were in sexually active age (15-50 years) attending Mehrad Hospital in Tehran, Iran, were collected from May 2009 to April 2010. Referrals were divided into two groups including symptomatic women (SW) with signs and symptoms referable to the urinary-genital tract and asymptomatic women (AW) attending hospital for nongenitourinary complaints.

Exclusion criteria included menstruation, pregnancy, consumption antibiotics in 3 past weeks, urogenital anomalies, kidney disease, catheter usage, cognitive impairments and severe diseases that precluded study interview.[8] All participants signed a consent form. The Research Ethics Board at Tehran University of Medical Sciences approved this study.

Ten ml of FVU were collected at least two hours after last urination in a sterile tube and biochemical analysis was done using standard Dipstick test.[9] Samples were maintained at 4°C for one night in order to decrease inhibitors in urine and then transported to the research laboratory on ice. Samples were centrifuged in 4°C (6000 g for 30 min) and pellets were washed with PBS (Phosphate buffer saline) twice and prepared for DNA extraction.[10]

Extraction was performed using DNG-Plus kit (Cinnagen, Tehran, Iran) based on following protocol: 100 µl of sample was mixed with 600 µl DNG solution in 1.5 ml Eppendorf tube and centrifuged at 7000 g for 5 min, then 400 µl isopropanol was added, centrifuged at 12000 g for 10 min. The pellets were precipitated with 1 ml of 70% ethanol and centrifuged at 12000 g for 5 min (precipitation was done twice). Poured off ethanol was allowed for air dry, finally DNAs were dissolved in 30 µl TE (Tris-EDTA) buffer and stored at -70°C until use for PCR.[11]

To establish condition for diagnostic procedure, we set up PCR using positive control DNA from C. trachomatis serovar L2 type strain 434/Bu (ATCC VR-902B). Amplification reactions were performed in a final volume of 20 µl containing 1 µl of positive DNA, 2 µl of the Master mix PCR solution (Bioatlas Mastermix, B.T. 10501) and 0.5 µl of each reverse and forward primer.

The primers KL1 (5´-TCCGGAGCGAGTTACGA AGA-3´) and KL2 (5´-AATCAATGCCCGGGATT GGT-3´) were used to amplify a specific 241 bp fragment of the C. trachomatis cryptic plasmid gene.[12]

The amplification was performed on a PCR system (TECHNE, FTC 51H2D). The reaction involved one cycle at 94°C for 5 min, followed by 30 cycles of denaturation at 94°C for 40 sec, annealing at 58°C for 45 sec and extension at 72°C for 40 sec, followed by one last elongation at 72°C for 8 min. The PCR products were analyzed by electrophoresis in the 0.5% TBE buffer through 1% agarose gel, stained with ethidium bromide (1ng/ml), and DNA bands were visualized using a UV transilluminator.

Data were analyzed using SPSS for Windows (Version 15, Chicago, IL, USA). To examine possible associations between a positive test and risk factors, a multivariate analysis was performed. All associations with a p value < 0.05 were as significant. Moreover, for showing the strength of association between variables, common odds ratio (%95 CI.) was estimated.

## Results

Of the 260 women recruited in this study, a high proportion of them were older than 30 years old (69.2% of SW and 63.0% of AW). Moreover, 73.0% of participants in each group were married and 45.3% of all had at least diploma or university educations (42.3% in SW and 48.4% in AW). Also in symptomatic group, dysuria and vaginal discharge were common symptoms consecutively.

**Figure 1:**
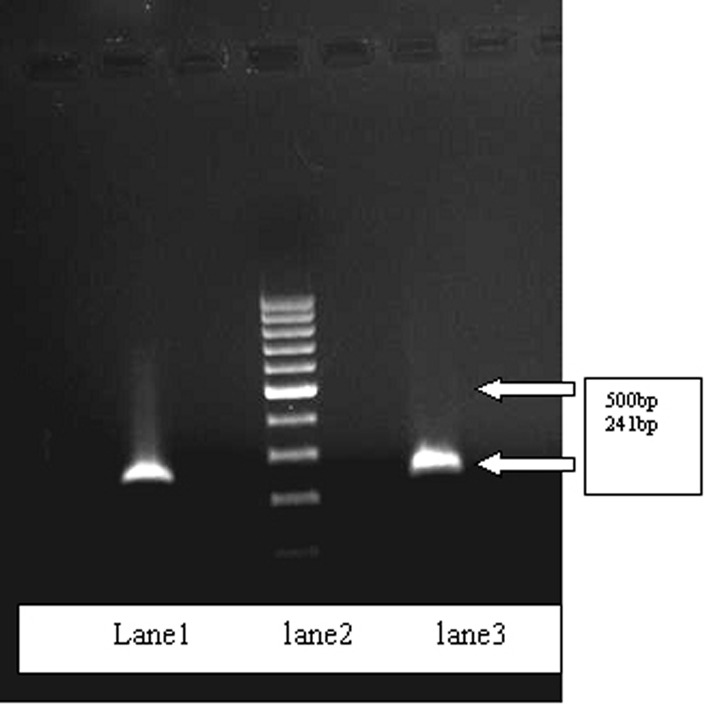
lane1: Control positive of C. trachomatis, Lane2: 100bp ladder, Lane3: positive sample.

Genital infections of C. trachomatis were detected in 39 out of 260 women in total (14.99%). There were 27/130 patients in symptomatic group (20.76%), compared with 12/130 patients in asymptomatic group (9.23%). No association was found between two groups. Data analysis showed infection with C. trachomatis in SW that was significantly associated with history of STIs, WBC in urine and epithelial cells in urine (p=0.05). Figure 1 shows the 241 bp amplified fragment of cryptic plasmid gene.

Table 1 illustrates statistical analysis results of important variables in women with positive C. trachomatis infection. In this table, history of STIs was seen in 29.2% of SW and 26.9% of AW in total. Also data analysis revealed that a high rate of white blood cells in urine of SW (81/130) and 37.6% of AW, In addition, about epithelial cells in urine, data showed 53.8% of SW and 43.0% of AW were in high range based on dipstick tests.

**Table 1:** Important variables in women with positive chlamydial infection.

**Cause**	**Yes**	**No**	***P* value**	**Odd ratio**
History of STIs				2.954
SW	15	12	≤0.05	
AW	4	8	NS^a^	
History of abortion				1.768
SW	13	14	NS	
AW	3	9	NS	
History of PTD				1.455
SW	7	20	NS	
AW	1	11	NS	
IUD Usage				1.510
SW	14	13	NS	
AW	4	8	NS	
WBC in urine^b^				
SW	9	18	≤ 0.05	
AW	4	8	NS	
Epithelial cells in urine^b^				
SW	7	20	≤ 0.05	
AW	6	6	NS	

^a^ Not Significant

^b^ Yes means High and No means few+ moderate in Dipstick test, symptomatic women (SW), asymptomatic women (AW).

## Discussion

There are few studies in Iran that report the prevalence of C. trachomatis from 14% to 24% in symptomatic women,[11] but some limitation of these studies should be mentioned for preventing bias. Firstly, the representative distribution of variables may be affected due to the small sample sizes of studies in the target populations. Secondly, the selection bias in participants who consecutively recruited from the selected clinics or venues that may limit the generalizability of the results. This likelihood was moderate by the chosen recruitment of participants in our study.

The reported prevalence of C. trachomatis infection is low in several Eastern Mediterranean countries such as the one report from United Arab Emirates with 2.6% prevalence rate.[12] In addition, a hospital based study from Jordan among symptomatic patients reported 4.6% prevalence rate,[13] and in another study that compared infertility women with control group, reported 3.9% and 0.7% respectively.[14]

Based on several studies conducted in developed countries, the prevalence of C. trachomatis infection varied with age, and young adult patients were considered a high-risk group.[15] In our study, high prevalence of C. trachomatis was seen in 30-50 years old women and odd ratio analysis revealed C. trachomatis infection in this group to be about 1.5 folds more than women under 30 years old (29/39 [OR=1.502]), and two previous studies which conducted in Islamic Republic of Iran confirmed our results.[16] Also, we found it remarkable that the prevalence rate among married women were almost two folds more than single women [OR=1.850], which the reason of this fact may be the result of social and cultural aspects and religious attitudes in Iranian society about legal marriage.

The significant association between C. trachomatis infection with history of STIs and WBC in urine which was seen in symptomatic women is similar to studies conducted in Iran and United States of America.[17][18] Decreased susceptibility to C. trachomatis infection in elderly has been attributed to epithelial changes in uterine surface,[19] and comparison in different age levels surveillance may result in this difference. According to previous data, C. trachomatis alters a variety of host cell responses including proinflammatory cytokine and chemokine secretion, inhibition of apoptosis, inhibition of IFN-α and inducible MHC class I and II expression.[20] Also, C. trachomatis infected cells may interfere with rapid and essential innate immune responses.[21] Although it can be related to first void urine, which contains a high level of PUS cells, but in symptomatic women, it was significant and may be important.

It is clear that partner announcement alone is not adequate for an optimal reduction in the C. trachomatis prevalence rate, and targeted screening programs in sexually active people are needed as part of case-finding strategy. In a new study, we evaluated the prevalence of C. trachomatis and Mycoplasma genitalium in pregnant women, which the results were 14.79% and 2.04% respectively, and it seems that more attention are needed in this group.[22] The programs should focus on repeated testing for individuals who are in high risk groups like as pregnant women and women who have had a history of STIs during the preceding years. In addition, we found it reasonable to recommend that all sexually active females with any genitourinary symptoms to be offered convenience screening for C. trachomatis infection firstly and all of married women behind that.

In order to optimize this strategy, sexual health services should become widely available, and the services should publicize in a comfortable and appealing way in country. However, asymptomatic C. trachomatis infected people have a key role in distribution of infection and may remain shadowy and therefore keep on spreading the infection to partners. Interventions of variable content may lead to favored changes in information, manner, awareness, self-efficacy, skills, and behaviors such as using appropriate procedures for contraceptive ways, sexually infection causes, and practice of protected sexual manners. Finally, further studies in order to determine the prevalence of infection and correlations between infecting people and other variables are needed in other cities of Iran.
